# Voluntary locomotor activity promotes myogenic growth potential in domestic pigs

**DOI:** 10.1038/s41598-018-20652-2

**Published:** 2018-02-07

**Authors:** Claudia Kalbe, Manuela Zebunke, Dorothea Lösel, Julia Brendle, Steffen Hoy, Birger Puppe

**Affiliations:** 1 Institute of Muscle Biology and Growth, Leibniz Institute for Farm Animal Biology (FBN), D-18196 Dummerstorf, Germany; 2 Institute of Behavioural Physiology, Leibniz Institute for Farm Animal Biology (FBN), D-18196 Dummerstorf, Germany; 3 Institute of Genetics and Biometry, Leibniz Institute for Farm Animal Biology (FBN), D-18196 Dummerstorf, Germany; 4 Department of Animal Breeding and Genetics, Justus Liebig University Giessen, D-35392 Giessen, Germany; 5 Behavioural Sciences, Faculty of Agricultural and Environmental Sciences, University of Rostock, D-18059 Rostock, Germany; 6 Institute of Livestock Farming, Mecklenburg-Vorpommern Research Center for Agriculture and Fisheries, D-18196 Dummerstorf, Germany; 7 Federal Office for Agriculture and Food (BLE), D-53179 Bonn, Germany

## Abstract

Self-determined physical activity is an essential behavioural need and can vary considerably between individuals of a given species. Although locomotion is suggested as a prerequisite for adequate function of skeletal muscle, domestic pigs are usually reared under limited space allowance. The aim of our study was to investigate if a different voluntary locomotor activity leads to altered properties in the muscle structure, biochemistry and mRNA expression of selected genes involved in myogenesis and skeletal muscle metabolism. Based on a video tracking method, we assigned pigs to three categories according to their total distances walked over five observed time points: long distance, medium distance, and short distance. The microstructure and biochemistry parameters of the *M. semitendinosus* were unaffected by the distance categories. However, we found distance-dependent differences in the mRNA expression of the genes encoding growth (IGF2, EGF, MSTN) and transcription factors (MRF4, MYOD). In particular, the IGF2/MSTN ratio appears to be a sensitive indicator, at the molecular level, for the locomotor activity of individuals. Our results indicate that the myogenic growth potential of pigs under standard rearing conditions is triggered by their displayed voluntary locomotor activity, but the covered distances are insufficient to induce adaptive changes at the tissue level.

## Introduction

Locomotion is a prerequisite for the development and adequate function of skeletal muscle tissue. In farm animals, skeletal muscle is of specific interest because it forms the basis of meat. Therefore, pigs were selected for intensified muscle growth over decades and approximately 50% of the body mass in pigs consists of musculature. On the other hand, domestic pigs are commonly reared under limited space allowance. The standard requirement of the floor area available for fattening pigs defined by the European Union is under 1 m^2^/pig (EU DIRECTIVE 2008/120/EC). In a previous study, we showed that the defined behavioural phenotypes regarding temperament and personality resulted in considerable differences in the exploratory behaviour and individual reactivity in pigs^1^. Nevertheless, detailed information about the distances covered by pigs under conventional rearing conditions is scarce and often incorrect. The main reason for the absence of accurate data seems to be difficulties in exactly quantifying the locomotion behaviour of pigs in a pen. As one methodological approach, the distances walked by pigs were scored by behavioural observations showing that increased space allowance may also increase the distances covered^2^. In contrast, health problems in sows (e.g., lameness) may lead to reduced locomotor activity^3^.

In recent years, different video tracking methods have been developed for the automatic or semiautomatic monitoring of locomotion, or moving activity, in pigs^4–7^. Brendle and Hoy^8^ introduced the VideoMotionTracker® as a software solution for tracking pigs on a video recording using the PC mouse or a touch-screen terminal to measure the distances that were covered. For example, the measured distances covered by individual fattening pigs during 24 h ranged from 1211 m at the beginning to 77 m at the end of the fattening period.

Physical exercise can generally influence muscle physiological characteristics by changing the muscle fibre type composition and capillarity. Studies on the effects of exercise have been mostly carried out with laboratory animals or humans. Only a few studies have investigated the influence of physical activity due to exercise or different rearing systems on skeletal muscle in farm animals such as pigs^9^. Petersen *et al*.^10^ found differences in the myofibre type profile regarding increased locomotive activity or exercise in pigs. In miniature pigs, an increased oxidative muscle capacity was found after treadmill exercise^11^. Further studies determined the effects of exercise level or expanded space allowance on the performance and meat quality of pigs^2,12–16^. Whether differences in voluntary locomotor activity in pigs reared under conventional conditions are reflected in muscular cellularity is not known. In addition, studies investigating the effects of physical activity in pigs below the sub-cellular level (i.e., on muscular gene expression and enzyme activities) are lacking.

The first step in our study was to assess the individual voluntary locomotor activity of domestic pigs kept in standard rearing conditions by a video-tracking method. Subsequently, we analysed the skeletal muscle tissue of 145-day-old pigs which had completed their ontogenetic hypertrophic growth^17^. Using this approach, our study aimed to investigate whether a different voluntary locomotor activity of the pigs (measured by the different distances walked by each individual) leads to altered properties in muscle structure and biochemistry and to modified expression of selected genes involved in myogenesis and skeletal muscle metabolism.

## Results

### Monitoring the individual voluntary locomotor activity of the focus animals

**Figure 1:**
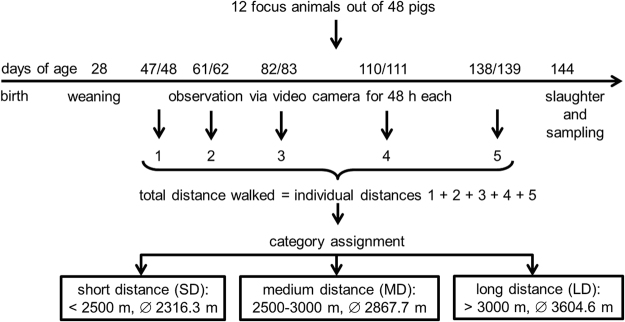
Experimental design for monitoring the individual voluntary locomotor activity of the focus animals. At five different ontogenetic time points the pigs were observed via video camera for 48 h each. The daily distances of each focus pig were analysed using the VideoMotionTracker® according to Brendle and Hoy^8^ and summed over the time points. All focus animals were assigned to the distance categories of short, medium or long distances (n = 4, each).

To determine the voluntary locomotor activity, we measured the daily distance walked by each of the 12 focus animals within 24 h in the home pen at five different time points during ontogenesis (Fig. 1). The 12 animals were assigned to three categories according their total distance walked: long distance (LD) (>3000 m, mean = 3604.6 m, n = 4), medium distance (MD) (between 2500 and 3000 m, mean = 2867.7 m, n = 4) and short distance (SD) animals (<2500 m, mean = 2316.3 m, n = 4). Moreover, we proved that the mean distance walked by the pigs within 24 h was affected by the distance category (LD: 720.9 ± 35.5 m, MD: 573.5 ± 35.5 m, SD: 463.3 ± 35.5 m; F_2,9_ = 13.27, P = 0.002). Pairwise comparisons revealed significant differences between the LD and SD animals (P = 0.002) and between the LD and MD animals (P = 0.040) but not between the MD and SD animals (P = 0.125).

**Figure 2:**
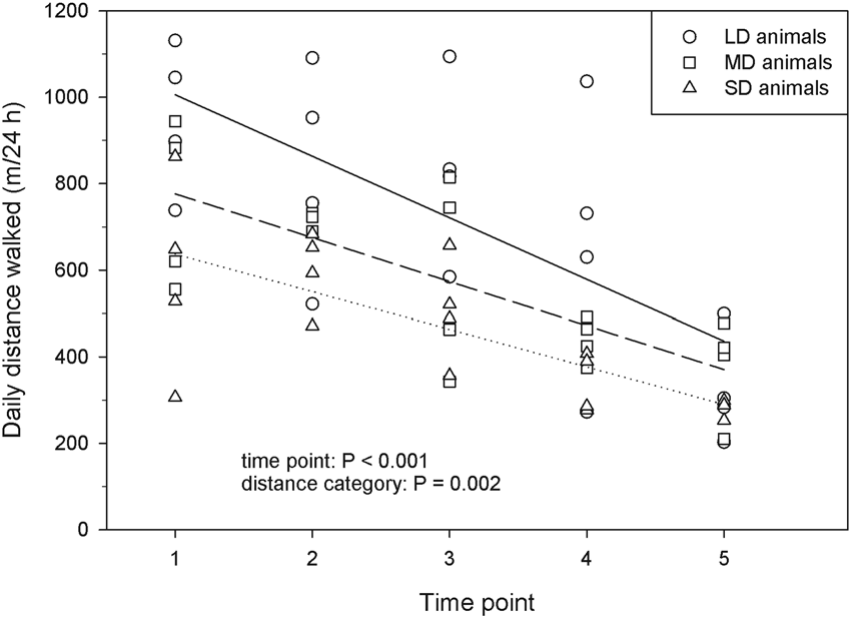
Development of the daily distance walked by the pigs for the three distance categories over five different time points (lsmeans, n = 12 focus animals; LD = long distance, MD = medium distance, SD = short distance). Lines indicate the calculated regression lines of each distance category over the different time points (solid line = LD animals: y = 1148.0x − 142.4, r² = 0.475; dashed line = MD animals: y = 878.0x − 101.5, r² = 0.560; dotted line = SD animals: y = 723.6x − 86.8, r² = 0.517).

The daily distances varied considerably between the observed individuals. The pig with the largest daily distance walked 1131.0 m at time point 1, the pig with the shortest distance walked only 202.4 m at time point 5 (Fig. 2). The daily distance walked was affected by the observed time point (F_4,36_ = 12.82, P < 0.001), showing a clear reduction in the distances with increasing age of the pigs. Pairwise comparisons showed a decrease from time point (TP) 1 (763.5 ± 49.2 m) and TP2 (712.9 ± 49.2 m) to TP4 (482.2 ± 49.2 m, P = 0.003 *vs*. TP1 and P = 0.019 *vs*. TP2, respectively) and TP5 (327.8 ± 49.2 m, P < 0.001 *vs*. TP1 and TP2, respectively) as well as from TP3 (643.1 ± 49.2 m) to TP5 (P < 0.001). The other pairwise comparisons (TP1 *vs*. TP2 (P = 0.952) and *vs*. TP3 (P = 0.446), TP2 *vs*. TP3 (P = 0.859), TP3 *vs*. TP4 (P = 0.177), and TP4 *vs*. TP5 (P = 0.209)) revealed no significant differences.

The weight of the animals was affected by the observed time point (F_4,36_ = 336.39, P < 0.001) but not by the distance category (F_2,9_ = 0.31, P = 0.741). The interaction of the time point × the distance category had no effect on the locomotor activity or the weight of the animals. In addition, there was no relationship between the total distances walked by the pigs and their body weights (see Supplementary Results, Table S1).

Taken together, our focus animals exhibited remarkable differences in their voluntary locomotor activity despite the limited space allowance under the rearing conditions used.

### Effects of total walking distance on muscular microstructure

To characterise the muscular phenotype of the pigs, several properties regarding myofibre size and type as well as skeletal muscle capillarity and intramuscular fat deposition were assessed after slaughter. The *M. semitendinosus* (ST) weight (LD: 346.8 ± 24.5 g, MD: 376.8 ± 24.6 g, SD: 369.3 ± 24.4 g; F_2,8_ = 0.40, P = 0.684) and the circumference (LD: 20.3 ± 0.8 cm, MD: 20.5 ± 0.8 cm, SD: 20.8 ± 0.8 cm; F_2,8_ = 0.12, P = 0.886) were not affected by the distance category. The relative area of intramuscular fat in the cross-section (LD: 2.7 ± 0.2%, MD: 2.5 ± 0.2%, SD: 2.4 ± 0.2%; F_2,8_ = 0.25, P = 0.787) was unchanged. The basic microstructural properties of ST muscle in terms of total fibre number (TFN) and fibre size (given as the fibre cross-sectional area, FCSA) of an average fibre, STO (slow twitch oxidative), FTO (fast twitch oxidative), FTG (fast twitch glycolytic), and pathologic fibre; relative fibre type distribution; the number of capillaries associated with one fibre and average fibre area supplied by one capillary did not differ among the distance categories, as shown in Table 1.

**Table 1:** Microstructural properties analysed in *M. semitendinosus* from focus pigs for the three distance categories (lsmeans ± SE, n = 12).

Property	long distance	medium distance	short distance	F	P
TFN (thousands)	953 ± 34	880 ± 34	895 ± 34	1.27	0.332
Capillaries/fibre	0.32 ± 0.06	0.43 ± 0.06	0.30 ± 0.06	1.46	0.287
Fibre area/capillary (µm²)	11,769 ± 1,491	9,890 ± 1,495	13,571 ± 1,483	1.52	0.275
FCSA (µm²)					
STO	4,049 ± 448	4,384 ± 449	4,887 ± 446	0.90	0.446
FTO	3,222 ± 337	4,036 ± 338	3,696 ± 336	1.45	0.291
FTG	3,468 ± 212	3,553 ± 213	3,627 ± 211	0.14	0.870
Pathologic	2,605 ± 609	1,678 ± 684	1,435 ± 706	0.88	0.464
Average	3,456 ± 269	3,847 ± 270	3,864 ± 268	0.73	0.511
Relative fibre number (%)					
STO	20.1 ± 2.6	22.2 ± 2.6	17.2 ± 2.6	0.90	0.443
FTO	28.1 ± 1.4	28.7 ± 1.4	29.5 ± 1.4	0.25	0.784
FTG	49.7 ± 1.8	47.9 ± 1.8	52.4 ± 1.8	1.53	0.273
Pathologic	2.2 ± 0.7	1.2 ± 0.7	0.9 ± 0.7	1.05	0.395

TFN – total fibre number; FCSA – fibre cross-sectional area; STO – slow twitch oxidative; FTO – fast twitch oxidative; FTG – fast twitch glycolytic.

A detailed analysis of the number of capillaries associated with each fibre type also revealed no differences among the distance categories (STO - LD: 0.55 ± 0.11, MD: 0.73 ± 0.11, SD: 0.52 ± 0.11; F_2,8_ = 1.14, P = 0.367; FTO - LD: 0.44 ± 0.09, MD: 0.56 ± 0.09, SD: 0.36 ± 0.09; F_2,8_ = 1.15, P = 0.363; FTG - LD: 0.17 ± 0.03, MD: 0.23 ± 0.03, SD: 0.18 ± 0.03; F_2,8_ = 0.93, P = 0.435; pathologic: LD: 0.25 ± 0.10, MD: 0.18 ± 0.11, SD: 0.09 ± 0.11; F_2,6_ = 0.54, P = 0.609). Likewise, the fibre area supplied by a single capillary showed no significant differences among the distance categories (STO - LD: 7738 ± 920 µm^2^, MD: 6614 ± 922 µm^2^, SD: 9595 ± 915 µm^2^; F_2,8_ = 2.69, P = 0.128; FTO - LD: 7995 ± 1368 µm^2^, MD: 8359 ± 1372 µm^2^, SD: 10,843 ± 1360 µm^2^; F_2,8_ = 1.30, P = 0.325; FTG - LD: 65,129 ± 19,862 µm^2^, MD: 13,397 ± 19,917 µm^2^, SD: 21,292 ± 19,752 µm^2^; F_2,8_ = 1.95, P = 0.204; pathologic: LD: 18,069 ± 8616 µm^2^, MD: 11,968 ± 8966 µm^2^, SD: 12,789 ± 12,196 µm^2^; F_2,4_ = 0.14, P = 0.870). Moreover, the microstructural muscle parameters showed no clear positive or negative correlations (see Supplementary Results, Table S2) with the total distance walked.

### Effects of total walking distance on muscular biochemistry and mRNA expression

In ST muscle, biochemical analyses were done to quantify the biological macromolecules DNA, RNA and protein contents and to determine the specific activities of the enzymes associated with muscle energy metabolism. Neither the total DNA, RNA and protein contents nor the specific activities of creatine kinase (CK, a marker for muscle differentiation), lactate dehydrogenase (LDH, a marker for anaerobic glycolytic energy production) and isocitrate dehydrogenase (ICDH, a marker for oxidative metabolic capacity) differed among the distance categories (Table 2, P > 0.05). Likewise, the biochemical parameters did not correlate positively or negatively with the mean total distance walked (see Supplementary Results, Table S3).

**Table 2:** Biochemical properties analysed in *M. semitendinosus* from focus pigs for the three distance categories (lsmeans ± SE, n = 12).

Property	long distance	medium distance	short distance	F	P
Total DNA (mg)	273.71 ± 19.01	293.31 ± 19.06	304.02 ± 18.90	0.66	0.544
Total RNA (mg)	165.08 ± 9.79	176.89 ± 9.81	167.82 ± 9.73	0.39	0.688
Total protein (g)	52.55 ± 3.39	55.67 ± 3.39	56.58 ± 3.37	0.39	0.690
ICDH (IU/g protein)	16.13 ± 1.10	15.75 ± 1.10	14.70 ± 1.09	0.46	0.648
LDH (IU/mg protein)	3.32 ± 0.13	3.42 ± 0.13	3.54 ± 0.13	0.70	0.523
CK (IU/mg protein)	26.11 ± 0.98	26.20 ± 0.98	26.63 ± 0.98	0.08	0.924

ICDH–isocitrate dehydrogenase, LDH – lactate dehydrogenase, CK – creatine kinase.

Total DNA, RNA, and protein = concentration × muscle weight.

**Figure 3:**
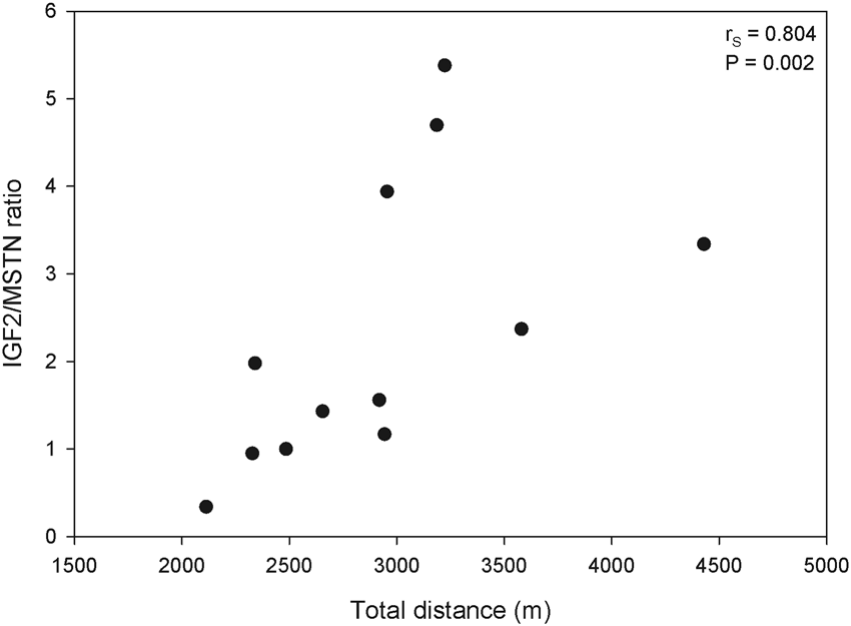
Relationship between the total distance walked by the pigs and the ratios between the mRNA expression of IGF2 and MSTN (black dots, n = 12 focus animals; IGF2 – insulin-like growth factor 2, MSTN – myostatin; r_S_ indicates the Spearman rank correlation coefficient and P the corresponding significance).

In the same muscle, mRNA expression levels of selected genes encoding myogenic transcription factors, growth factors and their corresponding receptors as well as proteins of muscle structure and metabolism were analysed. Interestingly, some genes encoding myogenic transcription and growth factors were differently expressed among the distance categories, while none of the genes associated with muscle structure or metabolism were affected (Table 3). The mRNA expression levels of growth factors MSTN, IGF2, and EGF were affected by the distance category (MSTN: F_2,8_ = 9.16, P = 0.009; IGF2: F_2,8_ = 5.63, P = 0.030; EGF: F_2,8_ = 4.52, P = 0.048). Pairwise comparisons revealed that the LD animals had a lower expression of MSTN (P = 0.007) and EGF (P = 0.050) than the SD animals. Moreover, for MSTN, the mRNA expression was reduced in MD animals compared with SD animals by a trend (P = 0.063). In contrast, the expression of IGF2 mRNA was increased in LD versus SD animals (P = 0.024). In addition, the IGF2/MSTN mRNA ratio was affected by the distance category (F_2,8_ = 5.96, P = 0.026). Post hoc tests revealed a significantly higher value in the LD group (3.90 ± 0.59) compared to the SD animals (1.06 ± 0.59, P = 0.022) with MD animals in between (2.08 ± 0.59, P = 0.140 *vs*. LD and P = 0.471 *vs*. SD, respectively). IGF1, AREG, BDNF, IGFBP5 and their corresponding IGF, EGF and GH receptors were unaffected by walking distance. Moreover, the transcription factors PAX7, MYF5, and MYOG remained unaltered with regard to the walking distance. MRF4 (F_2,8_ = 4.47, P = 0.049) and MYOD (F_2,8_ = 3.32, P = 0.089) mRNA expression differed or differed by trend, respectively, as a result of the walking distance. Animals from the MD category showed a lower MRF4 expression compared with the SD animals (P = 0.048), whereas the MYOD expression was increased by a trend between these two categories (P = 0.100). The Spearman rank correlation also showed significant interrelationships between the total distance walked and the IGF2, EGF, MSTN, and IGF2/MSTN ratios (Fig. 3). With increasing total distance walked, the specific mRNA expression of EGF (r_s_ = −0.627, P = 0.029) and MSTN (r_s_ = −0.783, P = 0.003) decreased, whereas the IGF2 mRNA expression (r_s_ = 0.713, P = 0.009) and IGF2/MSTN ratio (r_s_ = 0.804, P = 0.002, Fig. 3) increased. In addition, a reduction by trend of the MRF4 mRNA expression was found with increasing total distance walked (r_s_ = −0.553, P = 0.062). The other analysed genes revealed no positive or negative correlations with the total distance walked (see Supplementary Results, Table S4).

**Table 3:** mRNA expression of selected genes analysed in *M. semitendinosus* from focus pigs for the three distance categories (lsmeans ± SE, n = 12).

Gene	long distance	medium distance	short distance	F	P
myogenic transcription factors					
PAX7	1.14 ± 0.09	1.23 ± 0.09	1.03 ± 0.09	1.29	0.328
MYF5	0.99 ± 0.14	0.69 ± 0.14	0.78 ± 0.14	1.21	0.347
MYOD	1.66 ± 0.32 ^AB^	2.61 ± 0.32^A^	1.54 ± 0.32^B^	3.32	0.089
MYOG	1.26 ± 0.18	0.86 ± 0.18	1.17 ± 0.17	1.39	0.304
MRF4	0.65 ± 0.13^ab^	0.51 ± 0.13^a^	1.03 ± 0.13^b^	4.47	0.049
growth factors and growth factor receptors					
BDNF	0.43 ± 0.22	0.64 ± 0.22	0.67 ± 0.22	0.35	0.713
MSTN	0.36 ± 0.08^aAB^	0.53 ± 0.08^abA^	0.84 ± 0.08^bB^	9.16	0.009
IGF1	1.45 ± 0.17	1.65 ± 0.17	1.39 ± 0.17	0.65	0.546
IGF2	1.22 ± 0.09^a^	1.01 ± 0.09^ab^	0.80 ± 0.09^b^	5.63	0.030
EGF	0.52 ± 0.14^a^	0.63 ± 0.14^ab^	1.06 ± 0.14^b^	4.52	0.048
AREG	0.51 ± 0.24	0.42 ± 0.24	0.87 ± 0.23	1.03	0.401
IGFBP5	0.89 ± 0.12	0.82 ± 0.12	1.07 ± 0.12	1.07	0.389
IGF1R	1.00 ± 0.10	1.23 ± 0.10	1.14 ± 0.10	1.24	0.341
EGFR	0.82 ± 0.14	0.90 ± 0.14	1.02 ± 0.14	0.54	0.603
GHR	0.66 ± 0.09	0.80 ± 0.09	0.90 ± 0.09	1.70	0.242
muscle structure and metabolism associated genes					
PRKAA2	0.94 ± 0.14	1.04 ± 0.14	1.21 ± 0.14	0.96	0.424
SLN	0.89 ± 0.18	0.56 ± 0.18	0.91 ± 0.18	1.17	0.357
GATM	0.62 ± 0.21	0.66 ± 0.21	0.94 ± 0.20	0.71	0.522
CKM	0.90 ± 0.14	0.66 ± 0.14	0.75 ± 0.14	0.74	0.506
MYOT	0.90 ± 0.13	0.81 ± 0.13	0.85 ± 0.13	0.09	0.913
SORBS1	1.03 ± 0.19	0.95 ± 0.19	1.06 ± 0.19	0.10	0.908

Data are expressed as arbitrary units after normalisation by the endogenous reference gene HPRT1. AREG - amphiregulin; BDNF – brain derived neurotrophic factor; CKM - creatine kinase, M-type; EGF – epidermal growth factor; EGFR - epidermal growth factor receptor; GATM - glycine amidinotransferase; GHR – growth hormone receptor; IGF1, 2 – insulin-like growth factor 1, 2; IGFBP5 - insulin growth factor binding protein 5; IGF1R - insulin-like growth factor 1 receptor; MRF4 - muscle-specific regulatory factor 4; MSTN – myostatin; MYF5 – myogenic factor 5; MYOD - myogenic differentiation factor; MYOG – myogenin; MYOT - myotilin; PAX7 – paired box transcription factor 7; PRKAA2 - AMP-activated protein kinase catalytic sub-unit alpha-2; SLN – sarcolipin; SORBS1 – sorbin and SH3 domain containing 1.

Labelled least squares means within a row with different lower-case letters differ (P ≤ 0.05) or with upper-case letters tend to differ (P ≤ 0.10).

## Discussion

Locomotor activity, i.e., the active moving behaviour of an animal from one place to another, is an essential part of the movement ecology of the wild boar^18^. Wildlife studies using modern tracking technologies have reported considerable distances of several kilometres covered by some animals. For example, a 2-year old sow with her piglets covered a distance of at least 500 km within two months^19^. Reports on daily distances travelled by wild boars differ considerably in the literature^20–22^ and vary, for example, from 2.7 km to 6.8 km and partly more than 12 km depending on sex, age, season or available resources, but also affected by the animal habitat such as urban area or primeval forest.

However, domestic pigs kept in restricted housing conditions are clearly limited in their opportunities to display locomotor activity. Nevertheless, in our study, the measured daily distances covered by the pigs during 24 h showed their great need to perform locomotor behaviour. We found that the initially high daily voluntary locomotor activity decreased from time point 1 (763 m at age days 47/48) to time point 5 (328 m at age days 138/139) by more than half with increasing age. This confirms the locomotion data observed in our previous study^8^ under similar housing conditions. Moreover, the use of the VideoMotionTracker^®^ seems a suitable technique for phenotyping and quantifying the locomotion of livestock animals. We believe the present work is the first to demonstrate that pigs can be assigned to distance categories that significantly differ in their observed voluntary locomotor activity. For example, LD animals had a 56% higher voluntary locomotor activity than SD animals and a 26% higher locomotion level than MD animals. This suggests detectable individual differences in the internal motivation and appetitive drive to voluntarily perform locomotor activity. The question arises whether the differences are also linked to physiological properties of the skeletal muscle.

Generally, in humans or horses, it has been shown that exercise can influence skeletal muscle properties in terms of modified muscle fibre type composition and capillarity^23^. However, farm animals, such as pigs, are kept under an extremely limited space allowance without the opportunity for an exercise-like movement or even adequate physical activity.

We found no effects on live weight; therefore, it seems that the increase in the walked distance by up to 60% is not reflected in body mass of pigs. In addition, the absence of effects on the histological and histochemical properties of the *M. semitendinosus* in our study fits the results of Gentry *et al*.^2^ who studied increased exercise due to expanded space allowance in growing/finishing pigs without changes in muscular histology. In contrast, adaptations in muscle fibre characteristics induced by the spontaneous physical activity of pigs reared in large indoor pens and by treadmill training for 15 min per day were found by Petersen *et al*.^10^ in terms of a shift from IIB to IIA myofibres between trained and untrained pigs. We found no reduction in FTG myofibres (which are comparable to IIB), probably due to the limited space used in our study. Another explanation could be the possibility of performing more species-specific movements, such as the jumping or galloping of pigs, in larger pens than is possible under limited space allowance. Gondret *et al*.^9^ postulated that there generally seems to be a threshold for the induction of adaptive processes in muscle depending on the intensity of exercise and/or the specificity of the muscle. Therefore, we suggest that such a threshold was not met in our study or more generally under conventional rearing conditions for pigs. Consequently, an activity-induced increase in muscle fibre number was not present in our study. Besides, to our knowledge, an activity-induced increase in muscle fibre number has never been seen in other studies using pigs. The adaptation of fibre size was also not determinable in our study but was found by others (decreases^10^ or increases^16^). The lack of distance-associated effects on myofibre type distribution and fibre size seems to be the reason that the biochemical muscle properties (RNA, DNA, and protein content) and basic enzyme activities of muscle metabolism (ICDH, LDH, and CK) were also unaffected in our study. Therefore, we conclude that the existence of individual differences walked by pigs under conventional rearing conditions is not reflected in the structural and functional properties of the *M. semitendinosus*.

Although the total distances walked and/or differences between the distances are obviously too small to lead to adaptions in the skeletal muscle phenotype, we found distance-dependent differences in the mRNA expression levels of genes encoding muscle-associated growth factors and myogenic transcription factors. In contrast, the expression levels of genes associated with muscle structure or metabolism remained unchanged and fit the unaltered skeletal muscle cellularity of the pigs.

MYOD and MRF4 are muscle-specific transcription factors. In adult mammals, the mRNA expression of the myogenic regulatory factors results from satellite cells as myogenic stem cells, which are needed for muscle fibre hypertrophy or regeneration due to injury or exercise (reviewed in^24^). During myogenesis MYOD is important for myoblast proliferation and is also needed for cell cycle withdrawal^25^. MRF4 is involved in the maturation and maintenance of myofibres and the predominant expressed myogenic regulatory factor in adult myofibres^26^; however, the function of MRF4 in adult skeletal muscle is still unknown. We found decreased MYOD (by trend) and increased MRF4 mRNA expression in SD compared to MD pigs. Activated satellite cells exhibit a combined expression of PAX7 and MYOD^27^. However, a specific need for satellite cell activation due to hypertrophic growth or regeneration seems not to be required for the pigs in our study. The postnatal hypertrophic growth in pigs is normally finished at approximately 20 weeks of age^17^ and therefore, the pigs in our study with an age of 145 days should have finalised the increases in myofibre diameter and length. Moreover, as discussed above, the voluntary locomotor activity of our pigs can be viewed as only a basic level of movement under a limited space allowance compared to a real physical challenge; and therefore, there is no need for exercise-associated regeneration. Nevertheless, the pigs used the opportunity to move to different extents regarding their walking differences (approximately 2300 vs. 3600 m between SD and LD animals). A recent study^28^ provided evidence that MRF4 acts as a negative regulator of muscle growth. Therefore, the results for MRF4 and MSTN (or growth and differentiation factor 8, GDF8) – as a negative stimulator of skeletal muscle growth^29^ – are signs of impaired muscle growth in SD pigs. In humans, an early phase of muscle wasting due to 3 days of unilateral lower limb suspension was accompanied by an MSTN increase^30^.

In our study, the muscular mRNA expression of growth factor EGF was positively correlated with increased walking distance of the pigs. However, the mRNA expression of AREG, another ligand for the EGFR, or the mRNA expression of the EGFR itself was unaffected by the pigs’ walking distance. In general, EGF mRNA is known to increase with age in porcine skeletal muscle *in vivo*^31^ and from proliferation to differentiation in porcine primary cell cultures *in vitro*^32^. However, the specific role of EGFR ligands in skeletal muscle tissue growth is not clear. EGF was shown to stimulate skeletal muscle growth and differentiation *in vitro*^33–35^, whereas EGFR ligands also have the potential to decrease muscle mass and reduce body weights (reviewed in^36^).

The mRNA expression of growth factor IGF2 was negatively correlated with increasing walking distance of the pigs. IGF2 is regulated in a time-dependent manner and generally decreases with age in pigs^31^. It is commonly recognised that IGF2 (and IGF1) stimulate myoblast proliferation and differentiation *in vitro* (reviewed in^38^). In pigs a maternally imprinted (paternally expressed) mutation in the IGF2 gene resulted in increased IGF2 mRNA expression^38,39^, which was associated with intensified postnatal muscle hypertrophy due to a greater muscle fibre diameter and a higher proliferative capacity of satellite cells^40^. We could exclude that the IGF2 mRNA expression in our study was different due to the mutation because all pigs were tested and exhibited the same genotype. In addition, in previous experimental studies, we found increased muscular mRNA expression of IGF2 in 10-week-old piglets with a superior muscularity in terms of greater TFN and FCSA^41^ and decreased muscular mRNA expression in pigs with an opposite phenotype due to maternal protein reduction^42^. Therefore, we conclude that IGF2 mRNA expression reflects the muscular occurrence of pigs at postnatal ages. Moreover, a study^43^ found evidence that MSTN may negatively regulate IGF2 expression to control postnatal skeletal muscle growth in a study with MSTN-null and wild-type mice. The described relationship between MSTN and IGF2 expression fits our results. We found a significant correlation between MSTN (negative) or IGF2 (positive) and walking distance of the pigs. Furthermore, Lalani *et al*.^44^ postulated the ratio between IGF2 and MSTN mRNA expression as an indicator of the homeostatic balance that maintains skeletal muscle mass. In a spaceflight of rats, they found a low IGF2/MSTN mRNA ratio exhibiting the alterations of muscle homeostasis that favour loss in skeletal muscle. A comparable IGF2/MSTN mRNA ratio was also found in our SD pigs, although no signs of muscle loss were found in our histological or biochemical analyses. Therefore, the IGF2/MSTN mRNA ratio seems to be a highly sensitive indicator of the homeostatic muscle mass balance, which reflects changes in mRNA levels without phenotypic appearances. A few studies have also shown interactions between IGF2 and MYOD both in early differentiation *in vitro*^45^ and in corresponding mouse mutants *in vivo*^46^.

## Conclusions

We found clear individual differences in the motivation of domestic pigs to perform voluntary locomotor activity under standard rearing conditions with walking distances that differed by approximately 60%. These differences triggered differential mRNA expression, particularly of IGF2, MYOD, and MSTN in the *M. semitendinosus* and argue for a promotion of the myogenic growth potential in domestic pigs due to different walking distances. In particular, the IGF2/MSTN ratio seems to be a sensitive indicator at the molecular level for the pigs’ locomotor activity. In addition, the involvement of MRF4 and EGF is interesting because both are known as important transcription or growth factors in muscle tissue, but their specific function in adult skeletal muscle is unknown. However, there seem to be different thresholds for the induction of adaptive processes at the molecular and tissue levels. Potentially, due to the environmentally limited possibilities to act out the animals’ behavioural needs, the observed differences in the locomotor behaviour were insufficient to induce adaptive changes in the muscular phenotype, as characterised by muscle microstructure and biochemistry. Our study argues for a better understanding of the processes underlying the relationships between behaviourally induced physical activity and the phenotypic appearance of skeletal muscle in animals cared for and used by humans.

## Methods

### Animals, Housing and Husbandry

The study was conducted at the experimental pig unit of the FBN Dummerstorf, Germany. Animal husbandry and behavioural observations were done in accordance to national and international guidelines and approved by the institutional Animal Protection Board at FBN. In each of two replicates, 24 pigs (German Landrace) were used (n = 48; castrated males = 37, females = 11). At the age of 28 days, the piglets were weaned and transferred from the farrowing pens to another room containing a large pen (3.95 m x 4.45 m, 17.6 m²) with a partly slatted floor. The temperature of the room was adjusted to 25 °C after weaning and to 20 °C after the age of 70 days. Feeding occurred *ad libitum* at three feeding troughs with four feeding places in each (animal:feeding place ratio = 2:1). Water was available *ad libitum* at three nipple drinkers. Initially, the pen was reduced with metal bars to 9 m² allowing 0.38 m²/pig. At age day 67 the metal bars were removed and the space allowance was increased to 0.73 m²/pig. All space allowances provided met the standards for the protection of pigs both in Germany (TierSchNutztV, 2006, BGBL I, 2043) and the European Union (EU DIRECTIVE 2008/120/EC). At 144 days of age all animals were slaughtered after electro-stunning in the slaughterhouse belonging to the FBN and according to the current animal welfare regulations. The slaughterhouse is approved by the European Union and the German quality management system QS (MV21212). The focus animals were analysed in detail regarding the microstructure, biochemistry and mRNA expression of the *M. semitendinosus*. They were genotyped for the SNP (G3072A^38^) for the maternally imprinted *IGF2* gene as described previously^47^. All focus animals exhibited the A^pat^ genotype.

### Locomotion

After weaning, relocation and mixing, the animals were individually marked on their backs with an animal marker pen and observed via video camera (WV-BP 500 with wide-angle-lens: Panasonic TS3 V310) and infrared-technology (WFL-I/LED-30WN) for 72 h to determine the social rank structure. Six piglets per replicate were selected as focus animals (n = 12; males = 9, females = 3) according to their rank position, namely, animals with rank places 1 and 2 (high ranking), 11 and 12 (mid ranking), as well as 23 and 24 (low ranking). This approach was chosen to create balanced social focus groups for the observations. To determine individual locomotion behaviour, the twelve focus animals were observed via video camera at five time points (days 47/48, 61/62, 82/83, 110/111 and 138/139 of age) for 48 h each (Fig. 1). At each time point, the animals were weighed and the focus animals individually marked. The digitised videos were analysed for daily distances (individual distances walked by the focus animals within 24 h) with the VideoMotionTracker® according to Brendle and Hoy^8^. The daily distances of each animal were summed (total distances walked by the focus animals over the five observed time points) and ranked, and the 12 animals were then assigned to the three categories as described in the results section.

### Muscle microstructure

Within 10–15 min after slaughter, the entire ST was excised from the right hind limb and its weight, length and circumference were recorded. The muscle cross-sectional area (MCSA) was calculated from the circumference of the muscle mid-belly. Samples were snap-frozen in liquid nitrogen and thereafter stored at −80 °C. Serial transverse sections of 10 µm were cut at −20 °C in a cryostat microtome (Leica, Nussloch, Germany). One section was stained for cytoplasm with eosin and for alkaline phosphatase to visualise capillaries. Another section was exposed to the combined reaction for NADH-tetrazolium reductase^48^ and acid pre-incubated ATPase at pH 4.2^49^, which enables classification into STO, FTO, and FTG fibres. In addition, pathologic fibres (angular fibres and target fibres) were recorded. Fibre type distribution, FCSA, and capillary distribution were determined on 900 muscle fibres by image analysis (TEMA v1.00, Scan Beam APS, Hadsund, Denmark). Using the same software, the relative area of intramuscular fat in the cross-section was evaluated on oil red-stained sections over an area of 24 mm². The estimated TFN was obtained by multiplying the fibre number per unit area by the MCSA of ST muscle. All microscopic analyses were conducted by the same person.

### Biochemical analyses

A sample of 100 mg of muscle tissue was homogenised on ice in 2 ml of potassium phosphate buffer (pH = 6.9) using a Potter Elvehjem Tissue Grinder (Wheaton, Milleville, NJ, USA) and analysed for DNA, RNA, and protein concentrations and the activities of CK, LDH and ICDH. DNA were measured fluorometrically against a standard of calf thymus DNA (Sigma-Aldrich Chemie GmbH, Steinheim, Germany) after using Hoechst 33258 (Sigma-Aldrich)^50^. RNA was quantified fluorometrically with SYBR® Green II (Molecular Probes, Eugene, OR, USA) against RNA from calf liver (Sigma-Aldrich) as a standard^51^. In both assays, fluorescence was measured using an Flx-800-I microplate reader (Bio-Tek Instruments Inc., Bad Reichenhall, Germany). Protein concentration was determined^52^ against a bovine serum albumin standard (Serva, Heidelberg, Germany) and CK activity was measured in supernatants of diluted (1:10) muscle homogenates at 37 °C using a commercial kit (Biomed, Oberschleissheim, Germany). The activities of LDH and ICDH were measured using modified assay protocols according to SIGMA (http://www.sigmaaldrich.com/life-science/metabolomics/enzyme-explorer/learning-center/assay-library.html). All assays were adapted to microplates and the optical density was measured using a Spectramax Plus384 plate reader (Molecular Devices Corporation, Sunnyvale, CA, USA).

### RNA isolation, reverse transcription (RT) and qPCR

Total RNA was isolated from ST muscle tissue with the RNeasy fibrous Mini Kit (Qiagen, Hilden, Germany), as recommended by the supplier. This procedure includes the removal of genomic DNA with RNase-free DNase. The RNA was quantified in a NanoDrop instrument (Peqlab, Erlangen, Germany). Quality of the RNA was monitored using the Experion™ Automated Electrophoresis System (Biorad, München, Germany) in accordance with the manufacturer’s protocol. All samples were classified by an RNA quality indicator (RQI; 10 = intact RNA, 1 = highly degraded RNA) in the best category designation (7 < RQI ≤ 10).

RT was carried out with 2 µg of total RNA preparation, a mixture (2:1) of random primer p(dN)_6_ and anchored-oligo (dT)_18_ primer (Roche, Mannheim, Germany), and Moloney mouse leukaemia virus reverse transcriptase (M-MLV RT RNase H Minus Point Mutant, Promega, Mannheim, Germany) in 25 µl of the incubation buffer provided by the supplier, supplemented with deoxy-NTPs (Roche) and RNasin (Promega), for 60 min at 42 °C. The freshly synthesised cDNA samples were cleaned with the High Pure PCR Product Purification Kit (Roche) and eluted in 50 µl elution buffer.

For qPCR, 1.25 µl of each purified cDNA sample was amplified in duplicate with the LightCycler-FastStart DNA Master^PLUS^ SYBR Green I kit (Roche) in 10 µl of total reaction volume. Primer information is described in Table 4. All primers were purchased from Sigma-Genosys (Steinheim, Germany) and, if possible, were derived from different exons to avoid amplification of residual genomic DNA. Amplification and quantification of the generated products were performed in a LightCycler instrument 2.0 (Roche) under the following cycling conditions: pre-incubation at 95 °C for 10 min, followed by 40 cycles of denaturation at 95 °C for 15 s, annealing for 10 s at specific annealing temperatures (Table 4), extension at 72 °C for 10 s and single point fluorescence acquisition for 6 s to avoid quantification of primer artefacts. The melting peaks of all samples were routinely determined by melting curve analysis to ascertain that only the expected products had been generated. Additionally, the molecular sizes of the PCR products were monitored by agarose gel electrophoresis analysis. The relative quantification was performed with LightCycler software version 4.5 using the quantification module Relative Quantification – Monocolour. The relative expression ratio of the target gene is thereby calculated based on the PCR efficiencies and the crossing point deviation of an unknown sample *vs*. the calibrator (an arbitrarily selected sample) and expressed in comparison to an endogenous reference gene hypoxanthine phosphoribosyltransferase 1 (HPRT1) as previously described^53^. The HPRT1 expression was unaffected by the distance walked (F_2,8_ = 0.70, P = 0.523).

**Table 4:** Primers used for qPCR.

Gene	Accession no. or reference	Forward primer	Reverse primer	Size (bp)	T_A_ (°C)
AREG	NM_214376	GCCATTGCTGCTTTTGTCTCTGCC	TGGCAGTGACCCCGATCTGCT	198	60
BDNF	Solberg *et al*.^54^	AGCGTGTGCGACAGCATTAG	GTCCACTGCCGTCTTTTTATCC	60	58
CKM	Seale *et al*.^55^	GCAAGCACCCCAAGTTTGA	ACCTGTGCCGCGCTTCT	62	55
EGF	Kennedy *et al*.^56^	TCTGAACCCGGACGGATTTG	GACATCGCTCGCGAACGTAG	202	60
*EGFR*	Kalbe *et al*.^32^	TGGAGAAGCTCCCAACCA	CTCTTAATTCCTTGATAGCCACAG	161	60
GATM	NM_001128442	GGCAGCTTGAGATGTTGATCC	TCACCAGTGTTGAGATGAGAGT	256	60
GHR	Rehfeldt *et al*.^57^	TGATTCTACCCCCAGTTCCAGTTC	TCAGTCTTTTCATCAGGGTCATCA	187	56
HPRT1	Erkens *et al*.^58^	CCGAGGATTTGGAAAAGGT	CTATTTCTGTTCAGTGCTTTGATGT	181	59
IGF1	Kalbe *et al*.^32^	CTCTTCGCATCTCTTCTACTTGGC	CCTGTGGGCTTGTTGAAATAAAA	150	60
IGF2	Kalbe *et al*.^32^	TGGCATCGTGGAAGAGTG	AGGTGTCATAGCGGAAGAAC	164	57
IGFBP5	Rehfeldt *et al*.^57^	GTGTACCTGCCCAACTGTGA	AAGCTGTGGCACTGGAAGTC	158	56
IGF1R	Kalbe *et al*.^32^	GATTCAGGCCACCTCTCTCTCC	CCCTCCTACTATCAACAGAACGGC	139	60
MRF4	Maak *et al*.^59^	CGCCATCAACTACATCGAGAGGT	ATCACGAGCCCCCTGGAAT	189	60
MSTN	Maak *et al*.^59^	CCCGTCAAGACTCCTACAACA	CACATCAATGCTCTGCCAA	141	62
MYF5	Rehfeldt *et al*.^57^	CCTGAATGCAACAGCCCT	CGGAGTTGCTGATCCGAT	152	60
MYOD	Rehfeldt *et al*.^57^	GGTGACTCAGACGCATCCA	ATAGGTGCCGTCGTAGCAGT	108	60
MYOG	Rehfeldt *et al*.^57^	CAACCAGGAGGAGCGAGAC	AGGGTCAGCTGTGAGCAGAT	161	64
MYOT	NM_001099941	GGCTCGCAGATTGCTAGGACCA	GCTGTGGTGAATCTTGTGCGGC	83	60
PAX7	Patruno *et al*.^60^	CAACCACATCCGCCACAA	TCTTGGAGACACAGCCATGG	101	58
PRKAA2	Kalbe *et al*.^61^	TGACCCCCTGAAACGAGCAACTA	CAATGACAAGATGATAAGCCACTGC	233	55
SLN	NM_001044566	GGCACCCCATAGCACTTCTGACC	GGCAGCCCTTGAGAGCAGCAT	102	60
SORBS1	XM_001924661	CCACTGCAAGCCCTCAGCCTT	GTGACTCTTCGCTGCTGGGCT	106	65

T_A_, annealing temperature; AREG - amphiregulin; BDNF – brain derived neurotrophic factor; CKM - creatine kinase, M-type; EGF – epidermal growth factor; EGFR - epidermal growth factor receptor; GATM - glycine amidinotransferase; GHR – growth hormone receptor; HPRT1 - hypoxanthine phosphoribosyltransferase 1; IGF1, 2 – insulin-like growth factor 1, 2; IGFBP5 - insulin growth factor binding protein 5; IGF1R - insulin-like growth factor 1 receptor; MRF4 - muscle-specific regulatory factor 4; MSTN – myostatin; MYF5 – myogenic factor 5; MYOD - myogenic differentiation factor; MYOG – myogenin; MYOT - myotilin; PAX7 – paired box transcription factor 7; PRKAA2 - AMP-activated protein kinase catalytic sub-unit alpha-2; SLN – sarcolipin; SORBS1 – sorbin and SH3 domain containing 1.

To calculate the PCR efficiency, routine dilutions of the gene-specific external standards (cloned PCR products) of known concentrations covering five orders of magnitude (5 × 10^−16^ to 5 × 10^−12^ g DNA) were co-amplified during each run. Sequencing was performed with the automated sequencing system ABI PRISM 310 genetic analyser using the ABI PRISMBig Dye kit (both from PE Applied Biosystems, Weiterstadt, Germany).

### Statistical analysis

Statistical analyses were performed by analysis of variance (ANOVA) using the MIXED procedure of the SAS software for Windows, version 9.2 (SAS Institute Inc., Cary, NC, USA). The voluntary locomotor activity and the weight of the focus animals were analysed for the effects of the time points (1–5) and distance categories (LD, MD, SD) as fixed factors and their interactions, taking repeated measurements on the same individual into account. Data on the muscle structure, biochemistry and gene expression at slaughtering were analysed with distance category as the fixed factor and weight at slaughter as the co-variable. Pairwise multiple comparisons of the least-squares means were performed using Tukey-Kramer tests. Moreover, parameters measured at slaughter were correlated with the total distance walked by the pigs using the Spearman rank correlation (CORR procedure). Results were presented as lsmeans ± SE and were considered statistically significant when P ≤ 0.05 and were considered as tendencies when 0.05 < P ≤ 0.10.

### Data availability

The authors declare that all data supporting the findings of this study are available within the article and Supplementary Information, or are available from corresponding authors upon reasonable request.

## Electronic supplementary material

Supplementary Information
