# Supplementary material for: Voluntary locomotor activity promotes myogenic growth potential in domestic pigs
Source: Sci Rep. 2018 Feb 7;8:2533. doi: 10.1038/s41598-018-20652-2 (PMC5803246; doi:10.1038/s41598-018-20652-2)
Supplement: Supplementary file 1 — Supplementary Information [file 41598_2018_20652_MOESM1_ESM.doc]

### Supplementary Information

### Supporting Material for:

### Voluntary locomotor activity promotes myogenic growth potential in domestic pigs

### Claudia Kalbe^1^,*^,#^, Manuela Zebunke^2,3,#^, Dorothea Lösel^1,†^, Julia Brendle^4,‡^, Steffen Hoy^4^, Birger Puppe^2,5^,*

^1^Institute of Muscle Biology and Growth, Leibniz Institute for Farm Animal Biology (FBN), D-18196 Dummerstorf, Germany

^2^Institute of Behavioural Physiology, Leibniz Institute for Farm Animal Biology (FBN), D-18196 Dummerstorf, Germany

^3^Institute of Genetics and Biometry, Leibniz Institute for Farm Animal Biology (FBN), D-18196 Dummerstorf, Germany

^4^Department of Animal Breeding and Genetics, Justus Liebig University Giessen, D-35392 Giessen, Germany

### ^5^Behavioural Sciences, Faculty of Agricultural and Environmental Sciences, University of Rostock, D-18059 Rostock, Germany

* Corresponding authors.

[kalbe@fbn-dummerstorf.de](mailto:kalbe@fbn-dummerstorf.de) (C. Kalbe), [puppe@fbn-dummerstorf.de](mailto:puppe@fbn-dummerstorf.de) (B. Puppe)

^^These authors contributed equally.

^†^Present address: Institute of Livestock Farming, State Research Center of Agriculture and Fisheries Mecklenburg-Vorpommern, D-18196 Dummerstorf, Germany.

^‡^Present address: Federal Office for Agriculture and Food, Deichmanns Aue 29, D-53179 Bonn, Germany.

**Supplementary Table S1**

Relationship between the total distances walked by the pigs and the body weight (kg) of the focus animals

Property r_s_ P

Birth weight 0.538 0.071

Body weight at TP1 -0.284 0.372

Body weight at TP2 -0.189 0.557

Body weight at TP3 -0.256 0.422

Body weight at TP4 -0.287 0.366

Body weight at TP5 -0.238 0.457

Body weight at slaughter -0.161 0.618

r^s^ – Spearman-rank-correlation coefficient; P – significance value; TP – time point

**Supplementary Table S2**

Relationship between the total distances walked by the pigs and the microstructural properties of *M. semitendinosus*

Property r_s_ P

ST weight (g) -0.154 0.633

Circumference (cm) -0.137 0.672

Intramuscular fat (%) -0.077 0.812

TFN (thousands) 0.077 0.812

FCSA (µm²)

STO -0.252 0.430

FTO -0.357 0.255

FTG -0.147 0.649

Pathologic 0.309 0.385

Average -0.503 0.095

Relative fibre number (%)

STO 0.329 0.297

FTO -0.238 0.457

FTG -0.371 0.236

Pathologic 0.126 0.696

Capillaries/fibre 0.189 0.555

STO 0.035 0.914

FTO 0.273 0.391

FTG 0.179 0.577

Pathologic -0.207 0.567

Fibre area/capillary (µm²) -0.336 0.286

STO -0.175 0.587

FTO -0.413 0.183

FTG -0.084 0.795

Pathologic -0.048 0.911

## r^s^ – Spearman-rank-correlation coefficient; P – significance value; ST – *M. semitendinosus*; TFN – total fibre number; FCSA – fibre cross-sectional area; STO – slow twitch oxidative; FTO – fast twitch oxidative; FTG – fast twitch glycolytic

**Supplementary Table S3**

Relationship between the total distances walked by the pigs and the biochemical properties of *M. semitendinosus*

Property r_s_ P

Total DNA (mg) -0.378 0.226

Total RNA (mg) -0.021 0.948

Total protein (g) -0.203 0.527

ICDH (IU/g protein) 0.133 0.681

LDH (IU/mg protein) -0.483 0.112

CK (IU/mg protein) -0.196 0.542

r^s^ – Spearman-rank-correlation coefficient; P – significance value; ICDH – isocitrate dehydrogenase, LDH – lactate dehydrogenase, CK – creatine kinase

Total DNA, RNA, and protein = concentration × muscle weight

**Supplementary Table S4**

Relationship between the total distances walked by the pigs and the mRNA expression of selected genes in *M. semitendinosus*

Gene r_s_ P

myogenic transcription factors

PAX7 0.322 0.308

MYF5 0.245 0.442

MYOD 0.014 0.966

MYOG 0.179 0.578

growth factors and growth factor receptors

BDNF -0.196 0.542

IGF1 0.336 0.285

AREG -0.070 0.829

IGFBP5 0.084 0.795

IGF1R -0.229 0.473

EGFR 0.182 0.572

GHR -0.371 0.236

muscle structure and metabolism associated genes

PRKAA2 -0.098 0.762

SLN -0.189 0.557

GATM -0.203 0.527

CKM 0.063 0.846

MYOT -0.021 0.948

SORBS1 0.105 0.746

r^s^ – Spearman-rank-correlation coefficient; P – significance value; IGF1 – insulin-like growth factor 1; IGF1R - insulin-like growth factor 1 receptor; BDNF – brain derived neurotrophic factor; PRKAA2 - AMP-activated protein kinase catalytic sub-unit alpha-2, IGFBP5 - insulin growth factor binding protein 5, GHR – growth hormone receptor; PAX7 – paired box transcription factor; GATM - glycine amidinotransferase; MYF5 – myogenic factor 5; MYOD - myogenic differentiation factor; MYOG – myogenin; CKM - creatine kinase, M-type; EGFR - epidermal growth factor receptor; AREG - amphiregulin; SORBS1 – sorbin and SH3 domain containing 1; MYOT - myotilin; SLN - sarcolipin
